# Enhanced primers for amplification of DNA barcodes from a broad range of marine metazoans

**DOI:** 10.1186/1472-6785-13-34

**Published:** 2013-09-10

**Authors:** Jorge Lobo, Pedro M Costa, Marcos AL Teixeira, Maria SG Ferreira, Maria H Costa, Filipe O Costa

**Affiliations:** 1 IMAR-Instituto do Mar, Departamento de Ciências e Engenharia do Ambiente, Faculdade de Ciências e Tecnologia da Universidade Nova de Lisboa, 2829-516 Monte de Caparica, Portugal; 2 CBMA-Centro de Biologia Molecular e Ambiental, Departamento de Biologia, Universidade do Minho, Campus de Gualtar, 4710-057 Braga, Portugal

**Keywords:** Universal primers, DNA barcoding, Taxonomy, Marine metazoans

## Abstract

**Background:**

Building reference libraries of DNA barcodes is relatively straightforward when specifically designed primers are available to amplify the COI-5P region from a relatively narrow taxonomic group (e.g. single class or single order). DNA barcoding marine communities have been comparatively harder to accomplish due to the broad taxonomic diversity and lack of consistently efficient primers. Although some of the so-called “universal” primers have been relatively successful, they still fail to amplify COI-5P of many marine animal groups, while displaying random success even among species within each group. Here we propose a new pair of primers designed to enhance amplification of the COI-5P region in a wide range of marine organisms.

**Results:**

Amplification tests conducted on a wide range of marine animal taxa, rendered possible the first–time sequencing of DNA barcodes from eight separated phyla (Annelida, Arthropoda, Chordata, Cnidaria, Echinodermata, Mollusca, Nemertea and Platyhelminthes), comprising a total of 14 classes, 28 orders, 57 families, 68 genus and 76 species.

**Conclusions:**

These primers demonstrated to be highly cost-effective, which is of key importance for DNA barcoding procedures, such as for building comprehensive DNA barcode libraries of marine communities, where the processing of a large numbers of specimens from a wide variety of marine taxa is compulsory.

## Background

The so-called “universal” PCR primers are research tools of great utility for molecular ecological studies where the same locus is analysed across a broad range of taxa from different phyla. The universal primers designed by Folmer and colleagues [1] (HCO2198-LCO1490, henceforth named “Folmer primers”) for amplification of a 658 base pair (bp) fragment of the 5′ end of the mitochondrial gene cytochrome *c* oxidase subunit I (COI-5P), have shown to be very successful in the amplification of this gene fragment in a broad range of marine metazoan phyla. The Folmer primers have been probably the most widely used primer pair for amplification of COI in many animal groups in addition to marine organisms (3166 citations recorded in ISI, 25/10/2012) (e.g. [2,3]). Indeed, this was the primer pair selected by Hebert and colleagues for their proof-of concept study on Canadian moths, where they propose the DNA barcoding approach for species identification [4]. The mitochondrial DNA region delimited by Folmer’s primers (COI-5P) became the established DNA barcode region for animal life. With a growing number of studies attempting to examine DNA barcodes from different animal taxa, it quickly became apparent that the primer pair HCO2198 and LCO1490 was not so “universal” as thought before, as it would still fail to amplify some taxa.

Simison [5], for instance, verified that the Folmer primers and several combinations with degenerate primers were successful for only a small number of gastropod taxa. Also, Lohman et al. [6] tried to amplify COI-5P of perching birds (Aves: Passeriformes) with the universal LCO1490/HCO2198 primer pair, but the efforts were similarly unsuccessful, as they were with different combinations of primers. In another example, Blankenship et al. [7], failed to amplify COI-5P using the Folmer primers from the remnants of big-eye tuna (identified only through the mitochondrial 16S gene) inside the guts of deep-sea amphipods (*Scopelocheirus schellenbergi*). Also, the usefulness of the Folmer primers may be limited for decapod Crustacea because they are not optimized [8]. In echinoderms, amplification of COI is often challenging, either due to low amplification successor to the amplification of pseudogenes [9,10]. Given the vast geographic distribution and the important commercial value of holothurians, further work should focus on developing alternative primers for these species, because PCR (polymerase chain reaction) amplification could not be achieved with the primers available [11].

To overcome the limitations of the presumed universal primers, new primer pairs have been developed targeting specific large assemblages, such as birds [12], lepidopterans [13], or fish [14]. Still, the limited amplification success in some groups led to the development of alternative approaches, namely the design of degenerate primer pairs [5] or even primer cocktails [15]. The later approach has been very successful in COI-5P amplification in fish (e.g. [16]), although one of the primer cocktails was originally designed and tested in mammals, and the same occurred for the alternative primer pairs designed for birds [6] or Lepidoptera [13]. Despite the success of these group-specific PCR primers, to date no alternatives to Folmer primers have been proposed that are effective in a broad range of marine animals and particularly for marine invertebrates. Within the latter group, most PCR primers developed were phylum or class specific (see [17]), like primers designed for Echinoderms [10], Crustacea ( [18], D. Steinke unpublished in [19]), Gastropoda [20] and Annelids [21]. Here we propose a new pair of enhanced primers specifically designed to amplify the COI-5P barcode region from a broad taxonomic range of marine organisms. We compared its amplification potential with other broadly used primer pairs, and tested amplification success in 76 species from both vertebrates and invertebrates and a total of 8 animal phyla. The success of amplification in a broad-range of taxa indicates that these primers can be particularly valuable for building up reference DNA barcode libraries of complex marine communities.

## Methods

### Sample collections

Marine specimens were collected from various locations along the west coast of Portugal and preserved in 96% ethanol. A total of 130 specimens and 76 species belonging to 8 among the most common phyla of marine metazoa was identified by traditional methods based on morphological characters. The list of specimens investigated and respective collection data is displayed in Table 1.

**Table 1:** Taxonomic classification of the 76 species from marine eight metazoan phyla (plus one alga species) sequenced with the primers LoboF1 and LoboR1

**Species analyzed in this project**					**Species from the BLAST search**	
**Kingdom**	**Phylum**	**Class**	**Order**	**Genus/Species**	**Specimen / similarity (%)**	**GenBank Accession**
Animalia	Annelida	Polychaeta		*Axiothella constricta*	*Nicolea zostericola* / 80	HQ024409
				*Euclymene sp.*	Maldanidae sp. / 81	HQ023886
				*Euclymene robusta*	*Hyalinoecia sp*. / 79	GQ497524
				*Euclymene santandarensis*	*Axiothella rubrocincta* / 82	HM473326
				*Leiochone leiopygos*	*Axiothella rubrocincta* /83	HM473326
				Praxillella praetermissa	*Axiothella rubrocincta* / 80	HM473326
				Unidentified Orbiniidae	*Naineris laevigata* / 77	GU362690
			Sabellida	*Sabella pavonina*	*Branchiomma sp.* / 87*	ACF75771
			Spionida	*Scolelepis (Scolelepis) foliosa*	Spionidae sp. / 81	GU672502
			Phyllodocida	*Glycera alba*	*Glycera sp.* / 78	HM473389
				*Hediste diversicolor*	*Hediste diversicolor* / 98	FJ030974
	Arthropoda	Malacostraca	Decapoda	*Deosergestes corniculum*	*Eusergestes similis* / 85	DQ882149
				*Porcellana platycheles*	Anomura sp. / 87	HM464308
				*Pestarella tyrrhena*	Anomura sp. / 84	HM465098
				*Calappa granulata*	*Calappa granulata* / 99	JQ306054
				*Calappa pelii*	*Calappa granulata* / 99	JQ306054
				*Atelecyclus sp.*	*Cancer bellianus* / 86	JQ306131
				*Macropodia sp.*	*Macropodia rostrata* / 99	JQ306016
				*Pilumnus hirtellus*	*Pilumnus hirtellus* / 94	JQ306038
				*Carcinus maenas*	*Carcinus maenas* / 99	FJ581593
				*Lophozozymus incisus*	*Hermodice carunculata* / 98	AY495947
				*Pinnotheres pisum*	*Neosarmatium fourmanoiri* / 85	FN392165
				*Eualus cranchii*	Anomura sp. / 85	HM464348
				*Oplophorus spinosus*	*Oplophorus spinosus* / 100	JQ306166
				*Systellaspis debilis*	*Systellaspis debilis* / 99	JQ306181
				*Systellaspis pellucida*	*Systellaspis pellucida* / 99	JQ306183
				*Palaemon elegans*	*Palaemon elegans* / 99	JQ306030
				*Chlorotocus crassicornis*	*Chlorotocus crassicornis* / 100	JQ305891
				*Pandalina brevirostris*	*Eumunida capillata* / 82	EU243342
				*Plesionika acanthonotus*	*Plesionika acanthonotus* / 99	JQ306170
				*Plesionika heterocarpus*	*Plesionika heterocarpus* / 99	JQ306279
				*Stylopandalus richardi*	*Plesionika narval* / 99	JQ305932
				Unidentified Paguridae	*Emerita analoga* / 83	AF425302
			Amphipoda	*Ampithoe sp.*	*Stenothoidae sp* / 86	EF989710
				*Caprella andreae*	Amphipoda sp. / 82	GQ260853
				*Corophium sp.*	*Cerodontha incisa* / 80	EF104689
				*Elasmopus rapax*	Amphipoda sp. / 82	HM466480
				*Jassa sp.1*	*Jassa staudei* / 82	EU243782
				*Jassa sp.2*	*Jassa marmorata* / 83	EU243747
				*Maera inaequipes*	Amphipoda sp. / 82	HM465802
				*Melita palmata*	*Melita plumulosa* / 79	JN790072
				*Microdeutopus chelifer*	Amphipoda sp. / 83	HM466455
			Isopoda	*Idotea granulosa*	*Idotea baltica* / 90	FJ581714
				*Idotea pelagica*	*Idotea pelagica* / 98	JQ425512
				*Talitrus saltator*	*Pseudorquestoidea brito* / 91	JX094870
	Chordata	Ascidiacea	Stolidobranchia	*Microcosmus squamiger*	*Microcosmus squamiger* / 100	FJ528602
				*Styela clava*	*Styela clava* / 99	FJ528636
		Actinopterygii	Gobiesociformes	*Lepadogaster lepadogaster*	*Dormitator maculatus* / 82	AY722137
			Perciformes	*Pomatoschistus lozanoi*	*Pomatoschistus tortonesei* / 90	FJ751922
				Bass^γ^	*Dicentrarchus labrax* / 100	FN689114
				Tuna^γ^	*Thunnus albacares* / 99	GU451782
			Pleuronectiformes	*Solea senegalensis*	*Solea senegalensis* / 99	EU513739
	Cnidaria	Anthozoa	Pennatulacea	*Veretillum cynomorium*	Funiculina sp. / 96	JN227949
	Echinodermata	Asteroidea	Forcipulatida	*Marthasterias glacialis*	*Marthasterias glacialis* / 99	DQ077925
		Ophiuroidea	Ophiurida	*Ophiothrix fragilis*	*Ophiothrix fragilis* / 98	EU583122
		Echinoidea	Camarodonta	*Paracentrotus lividus*	*Paracentrotus lividus* / 99	EF462949
	Mollusca	Bivalvia	Nuculida	*Nucula sulcata*	*Nucula sulcata* / 100	DQ280017
			Arcoida	*Anadara sp.*	*Anadara diluvii* / 99	JF496763
			Mytiloida	*Mytilus edulis*	*Mytilus edulis* / 100	GU570521
			Pectinoida	*Anomia ephippium*	*Anomia sp.* / 70	GQ166573
		Cephalopoda	Myopsida	*Alloteuthis media*	*Alloteuthis media* / 99	EU668087
				*Loligo forbesi*	*Loligo forbesi* / 100	AF075402
				*Loligo vulgaris*	*Loligo vulgaris* / 99	AF075397
			Sepiida	*Sepia officinalis*	*Sepia officinalis* / 99	EF416306
			Octopoda	*Argonauta argo*	*Argonauta nodosa* / 99	AY557517
		Gastropoda		*Osilinus sauciatus*	*Osilinus sauciatus* / 100	JN686313
			Littorinimorpha	*Vermetus triquetrus*	*Peasiella mauritiana* / 80	HE590850
			Neogastropoda	*Nassarius reticulatus*	*Nassarius reticulatus* / 99	EU827201
				*Ocenebra erinaceus*	*Ocenebra erinaceus* / 100	AY995773
			Nudibranchia	*Armina maculata*	*Dermatobranchus sp.* / 85	HM162698
			Pleurobranchomorpha	*Berthella plumula*	*Bathyberthella antartica* / 84	AY345027
		Polyplacophora	Chitonida	*Acanthochitona crinita*	*Acanthochitona crinita* / 86	AF120627
				*Chaetopleura angulata*	*Chaetopleura angulata* / 100	AY377703
			Lepidopleurida	*Leptochiton algesirensis*	*Leptochiton algesirensis* / 93	HQ907849
	Nemertea	Enopla	Monostilifera	*Amphiporus sp.*	*Amphiporus sp.* / 89	EU255601
	Platyhelminthes	Rhabditophora	Polycladida	*Leptoplana tremellaris*	*Pseudostylochus intermedius* / 82*	BAB39492
Chromista	Ochrophyta	Phaeophyceae	Fucales	*Fucus spiralis*	*Fucus vesiculosus var. spiralis* / 100	EU646757

*Amino acid sequence search because poor information is available about these taxa, ^γ^indicates common name (muscle tissue was provided without morphological identification to the species level).

### DNA extraction

Muscle tissue was used to extract DNA from the specimens of all surveyed groups. DNA extracts were obtained using the E.Z.N.A. Mollusc DNA Kit (Omega Bio-tek), following the manufacturer’s instructions.

### Primer design

**Figure 1:**
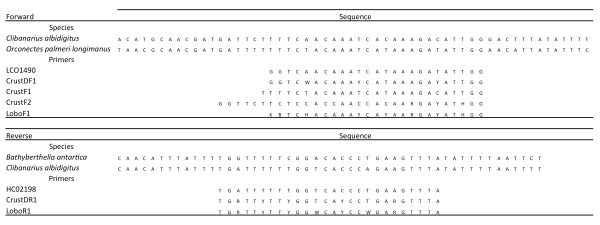
**COI sequences of three exemplificative species aligned with forward and reverse primers for the amplification of COI-5P.***Clibanarius albidigitus* (Phylum Arthropoda), GenBank Accession AF425321; *Orconectes palmeri longimanus* (Phylum Arthropoda), GenBank Accession AY701214; *Bathyberthella antarctica* (Phylum Mollusca), GenBank Accession AY345027. LCO1490 and HCO2198 [1]; CrustDF1 and CrustDR1 (D. Steinke unpublished in [19]); CrustF1 and CrustF2 [18]; LoboF1 and LoboR1 (present study).

Degenerate primers LoboF1 (forward) and LoboR1 (reverse) for the COI-5P were designed based on publically available COI sequences obtained from GenBank that were compatible with the COI–5P sequence that included complete 5′ and 3′ ends. They were analyzed and aligned using MEGA software version 5 [22]. To assist the design, three pairs of available primers were added to the alignment: the forward primers LCO1490 [1], CrustDF1 (D. Steinke unpublished in [19]), CrusF1 and CrusF2 [18] and the reverse primers HCO2198 [1] and CrustDR1 (D. Steinke unpublished in [19]) (Figure 1).

The amplifications were carried out in an iCycler™ (Bio-Rad) thermal cycler, using the new primers LoboF1 and LoboR1, and a pre-made PCR mix from Invitrogen™. The mix contained 1× PCR buffer, 1.5 mM of MgCl_2_, 0.2 mM of the dNTP mixture, 1 U of DNA Taq polymerase plus 0.5 μM of each primer and 4 μL of DNA template and completed with sterile milliQ-grade water to make up a total volume of 25 μL.

### DNA barcode amplification

The optimal annealing temperatures of primers were tested before setting up the PCR thermal cycling conditions based on the commonly used protocol in DNA barcoding studies: 1) denaturation at 94°C for 1 min; 2) denaturation at 94°C (30 s), annealing at 45°C (1 min 30 s), extension as 72°C for 1 min (5 cycles); 3) denaturation at 94°C (30 s), annealing at 54°C (1 min 30 s), extension as 72°C for 1 min (45 cycles) and ended with a final extension of 5 min at 72°C. The PCR products together with a 100 bp DNA ladder (Invitrogen™) were separated by electrophoresis in 1.5% agarose gel in TAE buffer, and subsequently stained with ethidium bromide for visualization in GelDoc 2000 equipment (Bio-Rad™). PCR products were cleaned up using a three-time precipitation with isopropanol, sequenced bidirectionally using the BigDye Terminator 3 kit, and run on an ABI 3730XL DNA analyser (all from Applied Biosystems™).

An initial test was conducted to compare the potential of newly-designed primers to amplify the COI barcode fragment, with two of the most common primer pairs used for marine organisms. The primers compared were LCO1490 and HCO2198, CrustDF1 and CrustDR1 and the new LoboF1 and LoboR1. DNA templates from six specimens of three crustacean species were tested in parallel for the 3 primer pairs employing the same PCR conditions.

### Sequence alignment and tree reconstruction

All sequence data were carefully checked to detect possible nuclear mitochondrial pseudogenes (numts) (see [23]): chromatogram quality examination and sequence editing to detect ambiguities, double peaks and noise, GenBank’s BLAST search [24] for homologies with public sequences (Table 1), and translation into amino acid for inspection for any indels, stop codons, or unusual amino acid sequence patterns. The GenBank accession numbers for sequences obtained in this study are included between KF369103 and KF369196, and specimen and sequence data is compiled in the Barcode of Life Data Systems (BOLD) project titled “Enhanced primers for amplification of DNA barcodes from marine metazoans”.

All sequence alignments and tree reconstruction were performed using MEGA5 software [22]. The neighbour-joining (NJ) method was used, applying the Kimura-2-parameter (K2P) model for the nucleotide–base tree [25] and the Jones-Taylor-Thornton (JTT) model for the amino acid sequence tree [26]. For assessment of node support 1000 bootstrap iterations [27] were run in both cases.

## Results

**Figure 2:**
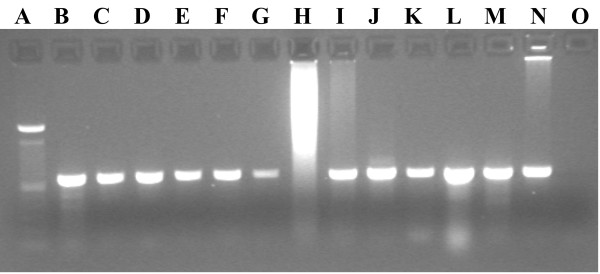
**Image an agarose gel of COI-5P PCR products amplified using the LoboF1 and LoboR1 primer pairs. A)** 100 bp Ladder, **B**-**G)** Class Bivalvia (Phylum Mollusca), **H**-**J)** Class Malacostraca (Phylum Arthropoda), **K)** Class Polyplacophora (Phylum Mollusca), **L)** Class Ophiuroidea (Phylum Echinodermata), **M)** Class Asteroidea (Phylum Echinodermata), **N)** Class Malacostraca (Phylum Arthropoda), **O)** Negative control. **H)** Possible excess of template blocked PCR amplification.

The primers LoboF1 and LoboR1 amplified the COI-5P (Figure 2) from a wide diversity of species from different animal phyla (Table 1), both vertebrates and invertebrates, comprising 130 specimens belonging to 76 species from 8 phyla. In addition, one brown alga species (*Fucus spiralis*) was successfully amplified (Table 1). All PCR products were successfully sequenced (forward and reverse sequences), with 87% of the cases returning the expected ≈ 658 bp length after sequence adition and primer trimming. No indication of the presence of numts was detected in any of the sequences, although the bivalve *Anomia ephippium* displayed two stop codons (TAA) on positions 3 and 158 of the amino acid sequence. This particular exception is further analysed in the Discussion section.

Contrasting the resulting sequences with those deposited at GenBank and BoldSystems databases confirmed the accordance between the morphological and genetic classification of most species. Although some specimens referred to species not yet accessioned in the databases, the specimen similarity obtained was always within the same taxonomic group, albeit at a higher taxonomic level.

**Figure 3:**
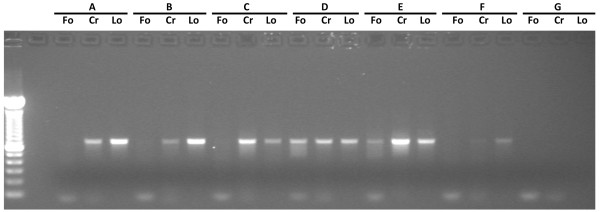
**Image of an agarose gel (1.5%) showing PCR products of crustaceans obtained with using different primer pairs. A)**, **B)** and **F)***Pilumnus hirtellus*; **C)** and **E)***Lophozozymus incisus*; **D)***Porcellana platycheles*; **G)** Negative control; Fo) LCO1490 and HCO2198 [1]; Cr) CrustDF1 and CrustDR1 (D. Steinke unpublished in [19]); Lo) LoboF1 and LoboR1 (present study).

Comparing the primers used in the initial test performed on six specimens, we verified that LoboF1 and LoboR1 were the only primers that provide DNA amplifications for all specimens (Figure 3). Folmer primers failed to amplify COI-5P for 4 out of 6 decapod samples and the crustacean-specific primers CrustDF1 and CrustDR1 amplified five of the six samples.

## Discussion

The newly-designed primers permitted amplification of COI-5P from all marine species tested, which comprised a wide phylogenetic diversity of taxa belonging to eight different phyla (Annelida, Arthropoda, Chordata, Cnidaria, Echinodermata, Mollusca, Nemertea and Platyhelminthes), and including some of the organisms considered more recalcitrant to amplification such as echinoderms and flatworms. To our best knowledge, this is the first DNA barcoding study reporting both successful amplification and sequencing from such a diverse array of taxa using a single pair of primers. Although the primers here proposed are degenerate, we did not find any indication of the presence of pseudogenes after detailed inspection of the sequences. The sequence chromatograms were generally of high quality, and neither indels or stop codons were observed, nor unusual substitution patterns in more conserved regions of the nucleotide or amino acid sequences. BLAST searches returned the expected matches or nearest neighbours, and we also did not find any evidence of accidental amplification of microbial contaminants (see [28]).

The only exception to this pattern was the presence of two stop codons (TAA) in the bivalve *Anomia ephippium*. Plazzi et al. [29] previously reported the occurrence of exactly the same stop codon (on position 158 of the amino acid sequence) on a specimen of the same genus (*Anomia sp*.), but still considered their sequence to have genuine mtDNA origin. Indeed, the chromatograms from our three specimens of *A. ephippium* had very high quality scores and did not show any other abnormal substitution except for these exact codons. Such a precise codon-specific substitution, occurring in three organisms of the same genus (diverging by 30%), should not be compatible with the expected random substitution pattern typical of a pseudogene (see for instance [23]). Similarly to Plazzi et al. [29], we suggest that this is a *bona fide* mtDNA COI sequence, and that the putative stop codon could be an exception to the mitochondrial code of this taxon that deserves further investigation.

Among the phyla that yielded successful COI sequences, echinoderms and flatworms (Platyhelminthes) have long been receiving particular attention in DNA barcoding and related studies due to acknowledged difficulties in obtaining quality sequences. Three species of three different classes of echinoderms had COI-5P successfully amplified and sequenced, albeit other authors acknowledge that amplifying COI-5P from echinoderms remains a challenge, probably due to the unsuitability of the primers used or to DNA degradation [10]. On the other hand, according to León-Règagnon et al. [30], the generation of primers that amplify the standard barcode region for a wide variety of flatworm groups is still at the experimental stage and the COI-5P traditionally used in platyhelminths is shorter than the standard barcodes. In the present study, only one species of platyhelminth (*Leptoplana tremellaris*) was attempted for amplification and sequencing, however, the standard COI-5P was successfully obtained, which may reveal a promising trend for the primers’ application to other flatworms. Additionally, even though the primer design was primarily aimed at invertebrate sequences, five distinct species of telosts (*Dicentrarchus sp.*, Perciformes: Moronidae, *Lepadogaster lepadogaster*, Gobiesociformes: Gobiesocidae, *Pomatoschistus lozanoi*, Perciformes: Gobiidae, *Solea senegalensis*, Pleuronectiformes: Soleidae and *Thunnus sp.*, Perciformes: Scombridae) were amplified and successfully sequenced. It should be noted, at this point, that several primer pairs [14] or even multiple primer cocktails [15] are usually used to amplify COI-5P. In spite of these promising preliminary results, research has still to be performed to confirm the widespread suitability of the LoboF1/R1 for teleosts.

Currently, COI-5P is a very useful marker to identify species and is generally regarded as a powerful tool for molecular taxonomy [4]. Furthermore, thanks to the BOLD multiple functionalities [31], sequences of COI-5P can already be contrasted with those introduced by other researchers. While COI-5P gives high support values for species identification, it does not resolve well deep molecular diversity, and shows a low level of phylogenetic informative characters [32], but the combination of nuclear and mitochondrial genes is useful for phylogenetic relationship (see [33,34]). Additionally, it must be stressed that DNA barcoding has not been technically conceived to recover phylogenetic relationships but rather to identify known species and to aid the discovery of new ones [35], which contributes to explain the misallocation of higher taxa hereby observed, in accordance to the observations by Hajibabaei et al. [36]. Still, regardless of technical limitations, all specimens of the same species were grouped in the same clade when either amino acid– or nucleotide–based trees were reconstructed, as expected.

## Conclusions

The newly designed primers LoboF1 and LoboR1 proved to be a rapid, practical and cost-effective tool for DNA barcoding based on COI sequencing. Given their high success rate to amplifying COI-5P from a wide and diverse range of tested taxa, it may well be that these new primers have a broader taxonomic range than all those currently used for barcoding the kingdom Animalia. These primers thus have high potential for the accelerated build–up of a global DNA–based biodiversity library, particularly with regard to the marine component.

## Competing interests

The authors declare that they have no competing interests.

## Authors’ contributions

JL participated in sample collections and morphological identifications, performed the DNA extraction, amplification and purification, designed the primers and wrote the manuscript. PMC provided support on molecular components, participated in the design of the primers and edited the manuscript. MALT and MSGF contributed on annelid and amphipod collections, morphological identifications, molecular and bioinformatic analysis. MHC participated in sample collections, morphological identifications and edited the manuscript. FOC provided supervision and training on molecular and bioinformatics components and edited the manuscript. All authors read and approved the final manuscript.
